# Safe but Lonely? Loneliness, Anxiety, and Depression Symptoms and COVID-19

**DOI:** 10.3389/fpsyg.2020.579181

**Published:** 2020-12-04

**Authors:** Łukasz Okruszek, Aleksandra Aniszewska-Stańczuk, Aleksandra Piejka, Marcelina Wiśniewska, Karolina Żurek

**Affiliations:** ^1^Social Neuroscience Lab, Institute of Psychology, Polish Academy of Sciences, Warsaw, Poland; ^2^Faculty of Psychology, University of Warsaw, Warsaw, Poland

**Keywords:** loneliness, mental well-being, anxiety and depression, COVID-19, risk perception

## Abstract

**Background:**

The COVID-19 pandemic has led governments worldwide to implement unprecedented response strategies. While crucial to limiting the spread of the virus, “social distancing” may lead to severe psychological consequences, especially in lonely individuals.

**Methods:**

We used cross-sectional (*n* = 380) and longitudinal (*n* = 74) designs to investigate the links between loneliness, anxiety, and depression symptoms (ADS) and COVID-19 risk perception and affective response in young adults who implemented social distancing during the first 2 weeks of the state of epidemic threat in Poland.

**Results:**

Loneliness was correlated with ADS and with affective response to COVID-19’s threat to health. However, increased worry about the social isolation and heightened risk perception for financial problems was observed in lonelier individuals. The cross-lagged influence of the initial affective response to COVID-19 on subsequent levels of loneliness was also found.

**Conclusion:**

The reciprocal connections between loneliness and COVID-19 response may be of crucial importance for ADS during the COVID-19 crisis.

## Introduction

Within 10 months’ time since the first case of the novel coronavirus originating from Wuhan (Hubei, China) has been officially reported, COVID-19 has spread to 214 countries and territories affecting over 35 million individuals and causing over 1,039,000 deaths as of 7th October (Dong et al., 2020). The characteristics of the virus, including high variance in presentation of symptoms, high transmission rates, a relatively long incubation period, and heightened mortality rates in elderly and individuals with pre-existing conditions (WHO-China Joint Mission, 2020), have led governments worldwide to implement unprecedented strategies to counteract its further spread. The outbreak was declared a pandemic by the WHO on March 11th, and as of early April, the largest increase in the daily number of cases has been observed in Europe and the United States (World Health Organization [WHO], 2020). Due to the exponential growth of COVID-19 cases observed across most EU countries, strategies aimed at “flattening the curve” by decreasing the number of simultaneous severe COVID-19 cases to a level that is manageable by the healthcare system were implemented at various paces by all EU countries. This includes Poland, which started introducing lockdown-type measures soon after the first death from COVID-19 in Poland on March 12th (Hirsch, 2020). On March 13th, the Polish government declared a state of epidemic threat and reinstated border controls; restricted the operation of shopping malls, restaurants, bars, and pubs; closed schools and universities; and banned public gatherings exceeding 50 individuals (Hirsch, 2020). Furthermore, citizens were recommended to work remotely if possible, engage in social distancing, and avoid leaving home unless necessary. Timeline of COVID-19 actions is visualized in Supplementary Figure 1. At the same time, no general lockdown was implemented at that point, and thus voluntary compliance from citizens was the driving factor for the effectiveness of the implemented strategy. Social distancing was sanctioned by law on March 24th, when gatherings of more than two people and non-essential travel were prohibited by law.

While crucial to limiting the spread of the virus, implementing necessary precautions to fight the pandemic inevitably results in a drastic suppression of direct interactions and a potential erosion of social bonds. Perceived social isolation, or loneliness, has been pointed out as one of the fundamental concerns during the current epidemiological crisis (Killgore et al., 2020a). At the same time, recent findings on the subject are mixed. Some studies provided evidence that perceived impact of the pandemic can actually mobilize individual social resources, sheltering one from the feeling of isolation and negative psychological outcomes (Luchetti et al., 2020; [Bibr B42][Bibr B57]). A study by McGinty et al. (2020) reported only a slight increase in loneliness during current events as compared with a survey conducted in 2018. At the same time, other research reveals a significant increase in declared loneliness after introducing stay-at-home policies (Killgore et al., 2020a, b), especially in the vulnerable groups (Bu et al., 2020) and young adults (Lee et al., 2020).

The possible impact of increased perceived social isolation during the current crisis is especially alarming from the perspective of the Evolutionary Theory of Loneliness (ELT; Cacioppo et al., 2006), which posits that prolonged loss of reliable social bonds can result in self-preservation bias and implicit vigilance toward threats. This in turn may provoke further disconnection from others and, in the longer term, can have a deleterious impact on mental and physical health (Holt-Lunstad et al., 2015). Loneliness also was found to predict higher stress appraisals (Hawkley et al., 2003) and increased threat perception (Qualter et al., 2013), making it plausible that lonely individuals may appraise the current outbreak situation more negatively and suffer from higher levels of distress. Indeed, a fast-growing literature on the impact of the current crisis on mental health provides evidence that loneliness constitutes a risk factor for distress, depression, and anxiety (e.g., Killgore et al., 2020a, b; Losada-Baltar et al., 2020; Palgi et al., 2020; Park et al., 2020; Tso and Park, 2020). Additionally, a recent review of the psychological effects of quarantine confirmed the potential severity of prolonged isolation (Brooks et al., 2020).

Importantly, while the link between mental health and loneliness has been reported repeatedly in recent research on the subject, the relationship between loneliness and the preventive strategies used in response to this epidemiological emergency is unclear. This issue is of particular importance during times of epidemiological emergency, when individual actions can have a critical effect on collective safety. While cognitive processes biased toward self-preservation (Spithoven et al., 2017) may be suboptimal during normal circumstances, the increased susceptibility to threatening aspects of the environment may contribute to implementing enhanced precautions against potential danger during a pandemic. At the same time, lonely individuals have been also shown to engage to lesser extent in health behaviors (Segrin and Passalacqua, 2010) and were found to exhibit less prosocial behavior (Twenge et al., 2007), and thus may be less willing to commit to self-imposed quarantine, especially in the absence of symptoms.

Studies on Ebola (Yang, 2016), H1N1 (Prati et al., 2011b), and SARS (de Zwart et al., 2009) showed that the perception of risks associated with each of the viral agents was one of the key factors driving societal response to their outbreaks. At the same time, it has been shown that affective response to a specific disease rather than cognitive evaluations of risks associated with it is crucial for one’s response to pandemic crisis (Prati et al., 2011b). Psychological characteristics, e.g., personality traits (Commodari, 2017), were found to shape individuals’ affective response to epidemics.

Limited data gathered during the current epidemiological crisis provide contradictory evidence. Wang et al. (2020) showed that access to reliable information and engaging in preventive measures were associated with less adverse psychological outcomes. A two-wave study conducted on Korean national representative sample revealed that despite accurate belief update of COVID-19 severity, participants were less willing to engage in preventive measures during the second wave of the study, and this decrease in motivation was mediated by increased depressive symptoms (Park et al., 2020). At the same time, later work on the subject revealed a positive association between depressive symptoms and more strict self-quarantine behavior (Nelson et al., 2020) and between stress and anxiety levels engagement in hygiene behaviors (Newby et al., 2020). Thus, it is still not clear to what degree adverse psychological symptoms are linked to precautionary behavior engagement and how the relation changes in time. Nonetheless, given the multitude of possible pathways linking loneliness and cognitive and affective factors associated with response to COVID-19 and self-isolation restrictions, loneliness may be among such characteristics.

Importantly, many studies on the impact of COVID-19 concerned older adults (e.g., Berg-Weger and Morley, 2020; Brooke and Jackson, 2020; Grossman et al., 2020; Parlapani et al., 2020; Patel and Clark-Ginsberg, 2020; van Tilburg et al., 2020). In this study, we decided to focus on young adults instead. It is believed that this group, while largely asymptomatic, may disregard restrictions and spread the virus (Kelly, 2020). Furthermore, this group is the least likely to perceive COVID-19 as a threat; a survey on a representative sample of adult Poles performed between March 5th and March 15th showed that almost 48% of respondents overall and more than half (58%) of participants aged 24–35 perceived the COVID-19 outbreak as “not special and was overblown by the media” (Pankowski, 2020). Finally, recent research showed that younger age is a risk factor for loneliness in general (e.g., Victor and Yang, 2012; Shovestul et al., 2020) and specifically during COVID-19 (Beam and Kim, 2020; Bu et al., 2020; Groarke et al., 2020; Li and Wang, 2020; Losada-Baltar et al., 2020; Luchetti et al., 2020). At the same time, it was pointed out that studies focused on this particular group are lacking (Groarke et al., 2020).

Given the importance of the initial response to the outbreak (Yeo and Ganem, 2020), our aim was to investigate the impact of early restrictions on appraisals and situational response during the early phase of the COVID-19 outbreak in Poland. Longitudinal research on the current situation is still scarce, yet crucial to disentangle the temporal dynamics of the psychological response (Groarke et al., 2020). Thus, the current study explored both cross-sectional and longitudinal relationships between loneliness, anxiety, and depression symptoms and compliance with recommended precautionary measures in a sample of young adults at two time points: immediately after restrictions were imposed upon population [3 days after the Polish government declared a state of epidemic threat and recommended social distancing (15th March)] and 2 weeks later, when the social distancing strategy was already sanctioned by law (29th March). We hypothesized that recommended restrictions might result in increased loneliness. At the same time, we posited a reciprocal association between feeling of isolation and mental health outcomes, such as individuals who are more lonely are also more prone to develop anxiety and depression symptoms, and initially poor mental well-being might contribute to the feeling of loneliness. As the literature on associations between loneliness, mental health, and preventive behaviors is mixed, we aimed at exploring whether and how worse psychological outcomes are linked to more precaution in the young adult population. Specifically, we wanted to investigate to what extent mental health and perceived social isolation were related to risk perception and affective response to the crisis among individuals who have followed social distancing recommendations.

## Materials and Methods

### Participants and Procedure

The initial (Wave 1; W1) online survey was performed via Qualtrics with an opportunity sample of individuals aged 18–35, who completed the open survey within a 36-h period starting at 9 PM on the 15th of March. The survey was distributed on Facebook groups, mostly devoted to student communities from different Polish universities and faculties. The survey was prepared in accordance with the Checklist for Reporting Results of Internet E-Surveys (CHERRIES) (Eysenbach, 2004). The questionnaire was distributed over 10 pages and consisted of 7 to 40 items per page. The survey was previewed by five researchers from our team. All questions had to be answered in order to submit the results and the participants could not change their answers after going to the next page of the survey. The participation rate for W1 was 0.93, while the completion rates were 0.56 and 0.45 for W1 and W2, respectively. Only completed questionnaires were analyzed. The IP address that appeared in the database more than once was checked in order to ensure that each entry contained a unique email address. It was the case for two duplicated IP addresses, and the entries were kept in the analyses.

The time constricted nature of the survey was utilized to grasp the immediate response to the restrictions introduced due to the state of epidemic threat, which had been declared 48 h prior to the start of the survey. The final sample consisted of 511 individuals (19% males, mean age: 23.3 ± 3.7) who were mostly students (77%) living in a large city (74%). Detailed demographic information is shown in Supplementary Figure 2. A follow-up survey was performed after a 14-day delay and started at 9 PM on March 29th. A group of 245 individuals who consented to be contacted again were invited to complete a follow-up survey via e-mail. One hundred ten participants who completed the follow-up until new restrictions were declared at 12 PM on 31st of March were included as Wave 2 (W2). Both surveys included Polish versions of standardized questionnaires measuring loneliness and anxiety and depression symptoms, as well as specific questions linked to the COVID-19 outbreak, which are described in detail below. The protocol of the study was accepted by the Ethics Committee at the Institute of Psychology, Polish Academy of Sciences. The participants were informed about the aim and length of the study and their right to withdraw at any moment prior to completing the survey. They were also told that the collected data will be anonymized and analyzed on the group level. Participants were not reimbursed for participation in the study.

### Loneliness and Anxiety and Depression Symptoms

The 20-item Polish version of the Revised UCLA Loneliness Scale (R-UCLA; Kwiatkowska et al., 2018) was used to measure loneliness. This adaptation of the R-UCLA consists of 20 items in the form of declarative sentences reflecting satisfaction and dissatisfaction with interpersonal relationships and was shown to have good test–retest reliability and external validity. Mental well-being was examined with the 30-item version of the General Health Questionnaire (GHQ; Frydecka et al., 2010). The Polish version of the GHQ-30 has excellent reliability (Cronbach’s α = 0.97) and was shown to have a three-dimensional structure. However, as items from the GHQ Social Relationships factor overlap thematically with the R-UCLA, and some of the GHQ General Functioning items could have been affected by objective restrictions (e.g., “Do you leave home as often as usual?”), we decided to utilize only the Anxiety and Depression subscale as the primary anxiety and depression symptoms (ADS) outcome.

### COVID-19 Items

The survey included specific questions about the level of impact of the COVID-19 pandemic on one’s daily functioning and professional activity, social context of self-isolation, and adherence to recommended preventive strategies. Furthermore, participants were asked to rate the perceived probability of various events associated with the COVID-19 outbreak (ranging from contact with a virus carrier to developing severe symptoms) and level of worry for 10 COVID-19-related issues on seven-point Likert scales from (1) *definitely not* to (7) *definitely yes*. During W2 the participants were additionally asked to rate their subjective complaints on 12 different issues associated with self-isolation. The items are presented in Table 1.

**TABLE 1:** COVID-19 items asked during W1 (A, B) and W2 (A, B, C).

Questions	Items
**Wave 1**	
**A) Affective response:** “To what extent, facing the SARS-CoV-2 pandemic in Poland, are you concerned about:”	(1) “Your health”
	(2) “The health of your loved ones”
	(3) “The ability of public healthcare to provide care to you and your loved ones”
	(4) “The ability of public healthcare to provide care to all members of society in need”
	(5) “The possible change in your financial situation”
	(6) “Access to the essential resources during the quarantine period”
	(7) “The condition of the economy”
	(8) “The loneliness and social isolation during the pandemic restrictions”
	(9) “The frustration and boredom caused by the pandemic restrictions”
	(10) “The lack of reliable information about the pandemic”
**B) Risk perception:** “How do you assess the likelihood of the following events occurring to you and your loved ones?”	(1) “Physical contact with an infected person”
	(2) “Being infected with the virus”
	(3) “Mild symptoms of the virus”
	(4) “Severe symptoms of the virus”
	(5) “Being hospitalized”
	(6) “Job loss”
	(7) “Loss of livelihood”

**Wave 2**	

**C) Subjective Complaints:** “To what extent in your daily life are you currently troubled by:”	(1) “Change of your daily routine”
	(2) “Inability to meet with family”
	(3) “Inability to meet with friends”
	(4) “Feeling of loneliness”
	(5) “Coronavirus news overload”
	(6) “Lack of reliable information on coronavirus”
	(7) “Limited access to various services and products”
	(8) “Boredom”
	(9) “Difficult contact with other people”
	(10) “Restricted freedom of movement”
	(11) “Feeling of uncertainty”
	(12) “Feeling of loss of control”

To avoid using single-item responses for further analysis, a principal component analysis with varimax rotation was performed on the scores of 511 participants separately for each variable. The subscales that have been created this way are described in detail in Supplementary Materials. Basic descriptive statistics and Cronbach’s alpha for each of the main W1 and W2 COVID-19 variables are shown in Table 2.

**TABLE 2:** Basic descriptive statistics and Cronbach’s alpha for each of the main W1 and W2 COVID-19 variables.

COVID-19 scales	Number of items	Mean (SD)	Cronbach’s alpha
**Wave 1**			
**Risk perception**	**7**	**3.44 (1.09)**	**0.80**
Contact risk	3	4.41 (1.37)	0.85
Severe symptoms risk	2	3.09 (1.48)	0.93
Financial problems risk	2	2.35 (1.61)	0.80
**Affective response**	**10**	**4.45 (0.95)**	**0.70**
Healthcare collapse worry	2	5.57 (1.45)	0.81
Isolation worry	2	3.61 (1.99)	0.83
Financial stability worry	3	4.29 (1.33)	0.57
Personal health worry	2	4.53 (1.45)	0.64
**Wave 2**			
**Subjective complaints**	**12**	**4.37 (1.28)**	**0.88**
Social isolation complaints (SIC)	7	4.30 (1.48)	0.87
Lack of control complaints (LCC)	3	4.62 (1.59)	0.75
Nonsocial deprivation complaints (NDC)	3	4.49 (1.44)	0.63

*Main outcome scores are presented in bold.*

### Statistical Analyses

Basic frequency statistics were calculated for variables linked to the impact of COVID-19 on participant functioning. Initially, we intended to use the level of adherence with COVID-19 preventive strategies during W1 and W2 as outcome variables in a path analysis. However, due to the extremely non-normal distribution of the strategies used [kurtosis: 3.68 (W1)/11.15 (W2)], it could not have been included in path models. However, to address the issue of preventive strategy use, we compared COVID-19 risk perception (W1), affective response (W1), and subjective complaints (W2) in participants who either complied (SDC) or did not fully comply (NSDC) with social distancing recommendation. SDC participants were defined as individuals who declared (1) avoiding direct social contact with others and (2) avoiding leaving the house unless necessary. Out of our initial sample of 511 individuals, 380 declared both, which, after exclusion of outliers (individuals with values over 1.5 interquartile range from first and third quartile of any of the variables included in the path model), left 366 SDC participants for path analysis. For W2 analysis, we included only participants who declared social distancing during both W1 and W2 (74 individuals), which left 69 SDC for our cross-lagged analysis after exclusion of outliers. SDC participants did not differ from NSDC (W1: *n* = 123; W2: *n* = 36): in terms of age, sex, education, work status, or place of residency. To examine W1 variables, we conducted a repeated-measures ANOVA with group (SDC vs NSDC) as a between-subject factor and risk perception (three levels) or affective response (four levels) subscales as within-subject factors. As *SIC* had twice as many items as the remaining two complaints subscales, separate between-group (SDC vs NSDC) *t*-tests were performed for each of the complaints subscales (Table 2). While path analysis allow for direct comparisons of path coefficients observed in SDC vs NSDC, we did not include NSDC participants as (1) the group who did not comply to social distancing measures was much smaller both at W1 and at W2 (W1: *n* = 123; W2: *n* = 36) and (2) NSDC could be further stratified into groups who did not comply with either (1) avoiding direct social contact with others or (2) avoiding leaving the house unless necessary, and (3) did not comply with both. Furthermore, NSDC included both participants who directly opposed regulations (e.g., 34% of NSDC who did not avoid social contacts) but also a significant group of respondents who were ambiguous about certain preventive strategies (e.g., 49% of NSDC when it comes to avoiding social contacts). Given that the prevalent majority of the participants could have been unequivocally classified as SDC we decided to rather drop the NSDC rather than draw any conclusions about the factors driving NSDC behavior during pandemics.

W1 model: Sequential mediation was tested by entering loneliness, anxiety and depression symptoms, risk perception, and affective response to COVID-19 to a path model in AMOS 25. Initial model was just identified; thus, after the initial step of the analysis, the non-significant paths were trimmed from them to enable examination of the model fit. Model fit was ascertained by using the chi-square statistic to examine the hypothesis that the matrix of the model parameters fits the observed covariance matrix. Additionally, goodness of fit was assessed by using the comparative fit index (CFI) and root mean square error of approximation (RMSEA) (Hu and Bentler, 1999). The significance of specific indirect pathways was examined by establishing whether 95% bootstrapped confidence intervals for each indirect effect contained the zero value.

W2 model: The cross-lagged effects between variables were investigated using a two-wave autoregressive cross-lagged panel model (CLPM), which allows one to examine directional effects of one variable on another over time, while accounting for the stability of each variable and their correlations at each time point. The longitudinal model was constructed on the basis of cross-sectional model W1, by including loneliness, anxiety, and depression symptoms, and COVID-19 affective response and risk perception observed during both waves in the CLPM. However, as no cross-lagged effects were observed for COVID-19 risk perception, it was removed from the model, thus leaving the three-variable CLPM. As two-wave cross-lagged models are just identified, testing of the model fit was not performed.

Only SDC without any missing data were included in W1 and W2 path analysis. Due to the time-constricted nature of the current study, we included all of the available observations without *a priori* power sample estimation. Retrospective analysis has shown that while all of our cross-sectional models were adequately powered, CLPM analysis could have been underpowered, especially when applying more stringent SEM assumptions (e.g., 20 subjects per variable assumption as recommended by Costello and Osborne (2005) would suggest that at least 120 participants should have been included in the analysis). Thus, the longitudinal analysis may have ignored the small correlations between variables.

## Results

### The Impact of COVID-19 on Functioning

Most of the participants considered their daily functioning (59%) and professional activity (80%) to be affected by the COVID-19 pandemic. The median predicted length of restrictions at the time of W1 was 31 days. Only 6% of participants reported spending the 2 weeks following W1 alone. The majority of the participants (86%) declared not being in a group particularly affected by COVID-19 (e.g., due to preexisting conditions); however, 75% of participants were directly linked to someone with increased risk to severe COVID-19 complications. The prevalent majority of participants declared using all of the recommended preventive strategies at W1 and W2 [washing hands and increased personal hygiene: 93% (W1)/93% (W2); avoidance of public places: 87%/92%; avoidance of public transportation: 77/92%; social distancing: 79/91%; leaving house only if necessary: 88/94%]. Social distancing became mandatory at the time of W2, which may partially explain the increase in strategy use. The only exception from high compliance was linked to wearing a mask, for which no clear recommendation was issued at either time point (9/35%). Use of preventive strategies was correlated with both loneliness (W1: *rho* = *-*0.20, *p* < 0.001) and COVID-19 affective response (W1: *rho* = 0.12, *p* = 0.007; W2: *rho* = 0.27, *p* = 0.005).

### Risk Perception and Affective Response in SDC and NSDC (W1)

SCD and NSDC groups did not differ in overall level of COVID-19 risk perception [*F*(1,487) = 1.54, *p* = 0.22, η_*p*_^2^ = 0.003, 95% CI = (0,0.021)] or in any specific domain [*F*(2,974) = 0.82, *p* = 0.44, η_*p*_^2^ = 0.002, 95% CI = (0,0.018)]. Overall, significant differences were observed in the perceived probability of the issues listed in each domain [*F*(2, 974) = 249.82, *p* < 0.001, η_*p*_^2^ = 0.339, 95% CI = (0.293,0.381)]. Participants perceived Contact Risk as rather likely (4.40 ± 1.34) and higher than Severe Symptoms Risk (3.07 ± 1.42; *p* < 0.001) and Financial Problems Risk (2.32 ± 1.56; *p* < 0.001). Severe Symptoms Risk was also deemed as more probable than Financial Problems Risk (*p* < 0.001).

No group differences were found in overall level of affective response to COVID-19 [*F*(1,487) = 3.01, *p* = 0.083, η_*p*_^2^ = 0.006, 95% CI = (0,0.027)]. Specific issues elicited various levels of affective response [*F*(3,1461) = 122.3, *p* < 0.001, η_*p*_^2^ = 0.201, 95% CI = (0.165,0.234)]. Participants were more concerned about Healthcare Collapse (5.62 ± 1.36) than of any other issue, while Isolation Worry elicited less affective response (3.60 ± 1.96) than other issues. With the exception of the difference between Financial Stability Worry (4.32 ± 1.26) and Personal Health Worry (4.56 ± 1.36), all of the remaining contrasts between categories were significant. An interaction between group and issue was also observed [*F*(3,1461) = 4.34, *p* = 0.005, η_*p*_^2^ = 0.009, 95% CI = (0.001,0.019)]: A higher level of Personal Health Worry was reported by SDC (4.71 ± 1.32) than by NSDC [4.11 ± 1.36, *t*(487) = 4.33, *p* < 0.001] participants, while no between-group differences were observed with regard to the remaining issues (Supplementary Figure 3).

### Cross-Sectional Model (W1)

**FIGURE 1:**
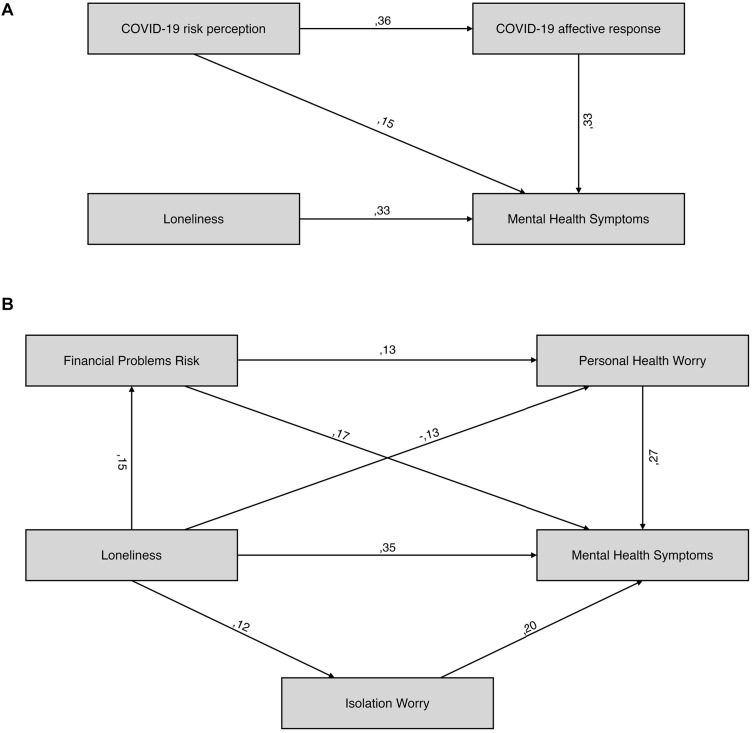
Cross sectional path models for the W1 loneliness, mental health symptoms and COVID-19 variables.

Prior to examining the path model, we examined zero-order correlations between loneliness, ADS, and COVID-19 risk perception and affective response in SDC (*n* = 366), both in general and in specific domains (Table 3). The final model, which is shown in Figure 1, had good fit to the data [χ^2^ (2) = 4.42, *p* = 0.11, RMSEA = 0.058, CFI = 0.986] and accounted for 28% of ADS variance. Similar effects of loneliness (β = 0.329, *p* < 0.001) and COVID-19 affective response (β = 0.335, *p* < 0.001) on ADS were found. COVID-19 risk perception was also a significant predictor of ADS (β = 0.152, *p* = 0.001). Furthermore, higher COVID-19 risk perception predicted a larger affective response to COVID-19 (β = 0.358, *p* < 0.001); thus, the total effect of COVID-19 risk perception on ADS (β = 0.270, 95% CI = 0.176 to 0.355, *p* = 0.001) was a sum of the direct effect (β = 0.152, *p* = 0.001) and indirect effect (β = 0.118, 95% CI = 0.073 to 0.175, *p* = 0.001) mediated through COVID-19 affective response.

**TABLE 3:** Zero-order correlations between loneliness, mental health symptoms and W1.

	(1)	(2)	(3)	(4)	4	(6)	(7)	(8)	(9)	(10)
Loneliness (1)	1									
MHS (2)	0.367***	1								
Risk Perception (3)	0.093	0.300***	1							
Affective Response (4)	0.088	0.413***	0.358***	1						
Contact Risk (5)	0.021	0.192***	0.823***	0.216***	1					
Severe symptoms Risk (6)	0.039	0.201***	0.715***	0.210***	0.454***	1				
Financial Problems Risk (7)	0.152**	0.271***	0.623***	0.364***	0.221***	0.179**	1			
Healthcare Collapse Worry (8)	−0.005	0.159**	0.231***	0.526***	0.217***	0.164**	0.111*	1		
Isolation Worry (9)	0.118*	0.251***	0.059	0.547***	0.044	0.006	0.074	−0.019	1	
Financial Stability Worry (10)	0.108*	0.268***	0.347***	0.714***	0.179**	0.096	0.490***	0.237***	0.137**	1
Personal Health Worry (11)	−0.114*	0.246***	0.268***	0.523***	0.162**	0.342***	0.107*	0.251***	0.003	0.248***

As no association between loneliness and general domains was observed, we also investigated the model that included specific domains, which have shown association with loneliness. The initial model included paths linking loneliness with ADS through Isolation Worry, Financial Stability Worry, Personal Health Worry, and Financial Problems Risk. Financial Problems Risk was also entered as a potential predictor of each affective subscale. Upon initial examination, Financial Stability Worry did not predict ADS and was excluded from the model. The new model had good fit [χ^2^ (2) = 1.22, *p* = 0.54, RMSEA < 0.001, CFI = 1] and accounted for 29% of ADS. Loneliness predicted higher Financial Problems Risk (β = 0.152, *p* = 0.003) and Isolation Worry (β = 0.118, *p* = 0.023), and lower Personal Health Worry (β = −0.133, *p* = 0.011). Each of the COVID-19 subscales also showed a significant relationship with ADS [βs ranging from 0.175 (Financial Problems Risk) to 0.267 (Personal Health Worry)]. Higher Financial Problems Risk also predicted larger Personal Health Worry (β = 0.128, *p* = 0.015).

The total effect of loneliness on ADS (β = 0.368) could be broken into the significant direct effect on ADS (β = 0.349, *p* < 0.001) and non-significant total indirect effects mediated by COVID-19 variables (β = 0.019, 95% CI = −0.024 to 0.070, *p* = 0.39). Interestingly, investigation of specific paths linking loneliness to ADS through COVID-19 variables revealed four significant indirect pathways. Positive mediations through Financial Problems Risk (β = 0.027, 95% CI = 0.007 to 0.057, *p* = 0.003) and Isolation Worry (β = 0.023, 95% CI = 0.003 to 0.052, *p* = 0.020) and double mediation through Financial Problems Risk and Personal Health Worry (β = 0.005, 95% CI = 0.001 to 0.014, *p* = 0.010) were found. However, a negative mediation of loneliness on ADS through Personal Health Worry was found (β = −0.035, 95% CI = −0.070 to −0.011, *p* = 0.010), which nullified the total indirect effect of loneliness on ADS.

### Subjective Complaints (W2)

Overall, each domain was seen as troubling (*SIC*: 4.30 ± 1.48; LCC: 4.62 ± 1.59; NDC: 4.50 ± 1.44) (Table 2). SDC participants reported more social isolation complaints compared to NSDC [*t*(103) = 2.29 SDC: 4.53 ± 1.35 vs NSDC: 3.85 ± 1.56, *p* < 0.05]. However, no significant differences were observed in the remaining subscales [LCC: *t*(102) = 1.84, *p* = 0.068; NDC: *t*(103) = 0.26, *p* = 0.80]. W2 loneliness was significantly correlated with *SIC* (*r* = 0.302, *p* = 0.012) and NDC (*r* = 0.253, *p* = 0.036) in SDC participants, but not in NSDC (SI: *r* = 0.173, *p* = 0.314; NDC: *r* = 0.052, *p* = 0.764).

### Longitudinal Model (W2)

Both ADS [W1: 21.7 ± 6.7 vs. W2: 23.4 ± 6.6, *t*(68) = 2.4, *p* < 0.05] and COVID-19 affective response [W1: 4.6 ± 0.6 vs. W2: 5.1 ± 0.8, *t*(68) = 6.3, *p* < 0.001] have increased between W1 and W2. Interestingly, no significant differences were found between W1 and W2 loneliness [W1: 39.8 ± 9.7 vs. W2: 41.0 ± 10.4, *t*(68) = 1.6, *p* = 0.12] and COVID-19 risk perception [1: 3.7 ± 1.0 vs W2: 3.7 ± 1.2, *t*(68) = 0.4, *p* = 0.71].

All of the auto-regressive effects were significant, with the most stable effects observed for loneliness (β = 0.726, *p* < 0.001) compared to ADS (β = 0.468, *p* < 0.001) and COVID-19 affective response (β = 0.516, *p* < 0.001). All of the correlations between W1 variables were significant (coefficients from 0.384 to 0.487, *p*s < 0.01). For the W2 variables, after controlling for their autoregressive and cross-lagged effects, only ADS remained significantly correlated with loneliness and COVID-19 affective response (coefficients of 0.432 and 0.385, respectively; *p*s < 0.01).

**FIGURE 2:**
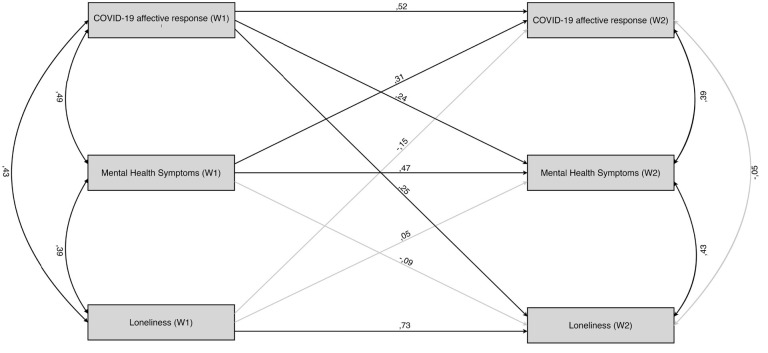
Cross-lagged panel model of the W2 variables. Nonsignificant paths are drawn in grey.

After controlling for the stability of the effects, no cross-lagged effect of loneliness on ADS or vice versa was found. Bidirectional cross-lagged effects between ADS and COVID-19 affective response were found, with higher ADS during W1 predicting larger COVID-19 affective response during W2 (β = 0.306, *p* = 0.004). However, a larger initial COVID-19 affective response also predicted higher ADS (β = 0.241, *p* = 0.031). Finally, the crossed-lagged effect of the initial COVID-19 affective response on loneliness levels during W2 (β = 0.251, *p* = 0.002) was found. The opposite cross-lagged effect of the initial level of loneliness on the COVID-19 affective response during W2 (β = -0.147, *p* = 0.156) was not significant, suggesting that the temporal effect of affective response to COVID-19 on loneliness is more robust than that of loneliness on COVID-19 response. The full CLPM model is shown in Figure 2.

## Discussion

Investigation of the factors shaping one’s response to the COVID-19 pandemic may be of crucial importance for developing response strategies aimed at mitigating the burden of prolonged self-isolation on well-being and mental health. Our study provides some initial insights into multiple possible links between loneliness, anxiety, and depression symptoms and response to the crisis. Importantly, only 6% of our participants declared spending their self-isolation period alone; thus, the observed mechanisms stem from the subjective appraisal of one’s social relationships, rather than objective social isolation *per se*. We observed that loneliness was correlated with ADS and with affective response to COVID-19’s threat to health. Furthermore, increased worry about the social isolation and heightened risk perception for financial problems was observed in lonelier individuals. The cross-lagged influence of the initial affective response to COVID-19 on subsequent levels of loneliness was also found. These findings will be discussed in detail below.

Firstly, as observed in our path analysis, loneliness may be linked to increased affective response to specific COVID-19 aspects while simultaneously being linked to decreased response to its other aspects. A similar magnitude of negative impact on anxiety and depression symptoms was found for loneliness and COVID-19 affective response in participants. Furthermore, COVID-19 risk perception increased the anxiety and depression symptoms of our participants both directly and by increasing their affective response to the situation, with both effects having a similar strength. Interestingly, no indirect effects of loneliness on anxiety and depression symptoms were found at the level of general indicators of COVID-19 affective response and risk perception. However, when specific issues were taken into consideration, loneliness predicted decreased affective response to COVID-19 as a threat to the personal health of our participants and of their close ones, increased affective response to potential detrimental effects of social isolation on social and psychological well-being, and increased risk perception of financial problems. However, when taken together, the specific trajectories (which were of opposite directions) canceled each other, which may explain the lack of indirect effects observed at the level of general indicators of COVID-19 response.

The fact that loneliness mitigated affective response to COVID-19 as a health threat may be linked to previous observations showing a negative association between loneliness and engagement in health behaviors (Segrin and Passalacqua, 2010), as many such behaviors are reinforced mostly by social support, participation, or inclusion, which lonely people are deprived of Hawkley et al. (2009); Shankar et al. (2011). Furthermore, the characteristics of our sample, with the majority of the participants not being in a special risk group (86%) but having someone from special groups among close ones (75%), suggest that this effect may be linked to reduced empathetic response to the potential health threat to others. Concern about COVID-19 health threats tends to increase together with perceived susceptibility of one’s family and friends (Wang et al., 2020; Zhu et al., 2020). Previous studies have shown that both trait loneliness (Beadle et al., 2012) and situational induction of loneliness (DeWall and Baumeister, 2006) are linked to decreased empathetic responding, which may have mitigated the affective response of a potential threat to the health of one’s close ones.

At the same time, loneliness was a predictor of response to secondary outcomes of the COVID-19 crisis, i.e., perception of risk of potential financial problems and stronger affective response to the impact of long-term isolation on psychological and social well-being. Previous research has shown that a proclivity for attaching high importance to money is higher in lonelier individuals, which has been suggested to be a safeguard against socioeconomic risks (Engelberg and Sjöberg, 2007).

Similarly, self-imposed quarantine can result in frustration, a deepening feeling of isolation, and boredom (Brooks et al., 2020). Thus, it is plausible that threats to economic and psychosocial well-being are more distressful for lonelier individuals than the direct impact of COVID-19 on physical health, as individuals already affected by the negative consequences of loneliness might experience the possibility of further disconnection as more distressing. This explanation is in line with recent research showing that, during the current crisis, individuals who are already lonely (Bu et al., 2020) and having less contact with relatives (Losada-Baltar et al., 2020) are more prone to loneliness and distress. It is also supported by the observation that loneliness is correlated with complaints of social isolation in participants who complied to social distancing guidelines for 2 weeks between W1 and W2. This was not the case in non-compliers. At the same time, we did not observe significant increase in loneliness *per se* during the 2-week period between W1 and W2 in participants. While surprising, this observation is congruent with Luchetti et al.’s (2020) study, which documented stable level of loneliness in a nationwide sample of American adults in late January/early February, late March, and late April 2020.

Interestingly, we did not observe cross-lagged links between loneliness and anxiety and depression symptoms, which would be expected on the basis of previous literature that observed reciprocal relationships between changes in loneliness and depressive symptomatology over 5-year (Cacioppo et al., 2010) or 14-year periods (Hsueh et al., 2019). However, as we found a stable relationship between anxiety and depression symptoms and loneliness measured at both time points, it is plausible that the time scale of the current study (2 weeks) was not suited for observation of longitudinal relationships between loneliness and anxiety and depression symptoms, which are observed with less time-constricted designs. These observations are also congruent with recent studies suggesting that loneliness is the main risk factor for depression, anxiety, and their comorbidity (Palgi et al., 2020), and loneliness may explain a significant portion of the variance of psychiatric symptoms observed in individuals during the COVID-19 crisis (Tso and Park, 2020). Furthermore, similarly to our findings, Killgore et al. (2020b) observed significant correlation between loneliness and both depression and suicidal ideation at all three data points of the study. Surprisingly, we observed the cross-lagged influence of initial COVID-19 response on subsequent levels of loneliness in social distancing individuals. While we rather expected to find the opposite relationship, this observation may be seen as a preliminary indicator of the deterioration of perceived social support due to disaster-related distress, which has been documented in studies on the psychological mechanisms observed in individuals suffering from disasters caused by natural hazards (Lai et al., 2018; Kaniasty, 2019). At the same time, bidirectional cross-lagged relationships between affective response to COVID-19 and anxiety and depression symptoms were found. Longitudinal analyses have shown that pre-event depressive symptomatology predicts post-event PTSD symptomatology in survivors of natural disasters (Ying et al., 2012). Thus, findings of the current study suggest that a similar reciprocal coupling between anxiety and depression symptoms and situational response to COVID-19 may be found even at the initial stages of response to COVID-19 pandemic. Similarly, Newby et al. (2020) has observed in a cross-sectional study with 5070 adult participants that participants with self-reported history of a mental health diagnosis had significantly higher distress, health anxiety, and COVID-19 fears than those without a prior mental health diagnosis.

Finally, we tentatively observed a negative link between loneliness and use of the recommended COVID-19 preventive strategies. The literature on people’s responses to public threat provides evidence that the feeling of belonging and affiliation is an important factor in shaping prosocial attitudes and behaviors (Lunn et al., 2020). Previous research demonstrated that empathic responders to the previous SARS outbreak were more likely to adapt effective and recommended precautions (Lee-Baggley et al., 2004; Puterman et al., 2009). Furthermore, the anxiety levels of family members and friends are related to affective response and implementing recommended behaviors (Prati et al., 2011a). Lack of important social bonds can therefore reduce one’s motivation to minimize the disease-related risk. In line with this notion, we found increased affective response to COVID-19 as a threat to health in individuals who voluntarily engaged in social distancing, before it was mandated by law (March 25th). Taking into consideration its significant negative association with loneliness, affective response to COVID-19’s threat to health may be a plausible mechanism mediating the relationship between loneliness and compliance with social distancing.

Given the mounting body of evidence that loneliness may be significantly associated with mental health outcomes, which includes both the current study and other studies carried out worldwide during the COVID-19 pandemic (e.g., Killgore et al., 2020a; Losada-Baltar et al., 2020; Palgi et al., 2020), this issue should be addressed while planning the interventions aimed at reducing the psychological burden of the pandemic. This may be particularly important, given the fact that a second wave of lockdown-like measures has started to be introduced in September 2020. Even though the nature of forced social distancing limits the possibility to mitigate objective social isolation, the evidence that objective and perceived social isolation are, to some extent, independent of each other has been presented (Cacioppo et al., 2014), which creates the opportunity to target loneliness via psychosocial interventions, even under lockdown-like measures. Moreover, it has been shown that interventions that target maladaptive social cognition are more successful in reducing loneliness than interventions that enhance social support or increase opportunities for social contact (Masi et al., 2011), which leaves an opportunity for addressing the issue of loneliness even under the conditions of social distancing.

### Limitations

While informative, our study was largely preliminary and opportunistic given the unpredictable time course of COVID-19 restrictions. Due to the use of computer-assisted web interviews, its population was limited to young adults and could not target elderly populations particularly prone to COVID-19. However, given the nature of the current crisis, a focus on young adults may be seen as both the limitation and strength of the study. Similarly, the time course of the study was correlated with the Polish timeline of the COVID-19 pandemic situation, and thus, the presented mechanism may vary depending on the pace and nature of the restrictions introduced by governments worldwide. Furthermore, with observational data, no causal relationship can be established. Finally, as we did not provide any reimbursement to participants, response rate at W2 was at 45%; this limited the statistical power of our analyses.

## Data Availability Statement

The datasets presented in this study can be found in online repositories. The names of the repository/repositories and accession number(s) can be found below: https://osf.io/ec3mb/.

## Ethics Statement

The studies involving human participants were reviewed and approved by Ethics Committee at Institute of Psychology, Polish Academy of Sciences. Written informed consent for participation was not required for this study in accordance with the national legislation and the institutional requirements.

## Author Contributions

ŁO developed the study concept. AP prepared the online survey. AP, MW, AA-S, KŻ, and ŁO performed the data collection and preprocessed the data. ŁO performed the data analysis and interpretation. MW prepared data visualizations. All authors contributed to the study design and contributed to the first version of the manuscript and approved the final version of the manuscript for submission.

## Conflict of Interest

The authors declare that the research was conducted in the absence of any commercial or financial relationships that could be construed as a potential conflict of interest.
