# Supplementary material for: Safe but Lonely? Loneliness, Anxiety, and Depression Symptoms and COVID-19
Source: Front Psychol. 2020 Dec 4;11:579181. doi: 10.3389/fpsyg.2020.579181 (PMC7747668; doi:10.3389/fpsyg.2020.579181)
Supplement: Supplementary file 1 [file Data_Sheet_1.docx]

**Checklist for Reporting Results of Internet E-Surveys (CHERRIES)**

| ***Checklist Item*** | ***Explanation*** | ***Page Number*** |
| --- | --- | --- |
| Describe survey design | The initial (Wave 1; W1) online survey was performed via Qualtrics with an opportunity sample of individuals aged 18-35, who completed the survey within a 36-hour period starting at 9 PM on the 15th of March. One-hundred ten participants who completed the follow-up until new restrictions were declared at 12 PM on 31st of March were included as Wave 2 (W2). | 5 |
| IRB approval | The protocol of the study was accepted by the Ethics Committee at the Institute of Psychology, Polish Academy of Sciences. | 6 |
| Informed consent | The participants were informed about the aim and length of the study and their right to withdraw at any moment prior to completing the survey. They were also told that the collected data will be anonymized and analyzed on the group level. | 6 |
| Data protection | The data collected in the project were fully anonymous and analyzed on the group level. | 6 |
| Development and testing | The survey was previewed by five researchers from our team. | 5 |
| Open survey versus closed survey | Open survey | 5 |
| Contact mode | Internet | 5 |
| Advertising the survey | The survey was distributed on Facebook groups, mostly devoted to student communities from different Polish universities and faculties. | 5 |
| Web/E-mail | a web survey | 5 |
| Context | The survey was distributed on Facebook groups, mostly devoted to student communities from different Polish universities and faculties. | 5 |
| Mandatory/voluntary | Voluntary | 5 |
| Incentives | No incentives were offered to the participants. | 6 |
| Time/Date | The initial (Wave 1; W1) online survey was performed via Qualtrics with an opportunity sample of individuals aged 18-35, who completed the survey within a 36-hour period starting at 9 PM on the 15^th^ of March.  A follow-up survey was performed after a 14-day delay and started at 9 PM on March 29^th^. One-hundred ten participants who completed the follow-up until new restrictions were declared at 12 PM on 31st of March were included as Wave 2 (W2). | 5, 6 |
| Randomization of items or questionnaires | NA |  |
| Adaptive questioning | NA |  |
| Number of Items | from 7 to 40 items per page. | 5 |
| Number of screens (pages) | 10 | 5 |
| Completeness check | Yes, all questions had to be answered in order to submit the results. | 5 |
| Review step | The participants could not change their answers after going to the next page of the survey. | 5 |
| Unique site visitor | NA |  |
| View rate (Ratio of unique survey visitors/unique site visitors) | NA |  |
| Participation rate (Ratio of unique visitors who agreed to participate/unique first survey page visitors) | W1: 914 (agreed to participate) / 986 (visited the first page/informed consent) = 0.93 | 5 |
| Completion rate (Ratio of users who finished the survey/users who agreed to participate) | W1: 511 (finished the survey) / 914 (agreed to participate) = 0.56  W2: 110 (finished the survey) / 245 (agreed to participate) = 0.45 | 5 |
| Cookies used | NA |  |
| IP check | The IP address that appeared in the database more than once was checked in order to ensure that each entry contained an unique email address. It was the case for two duplicated IP addresses and the entries were kept in the analyses. | 5 |
| Log file analysis | NA |  |
| Registration | NA |  |
| Handling of incomplete questionnaires | Only completed questionnaires were analyzed | 5 |
| Questionnaires submitted with an atypical timestamp | NA |  |
| Statistical correction | NA |  |

This checklist has been modified from Eysenbach G. Improving the quality of Web surveys: the Checklist for Reporting Results of Internet E-Surveys (CHERRIES). J Med Internet Res. 2004 Sep 29;6(3):e34 [erratum in J Med Internet Res. 2012; 14(1): e8.]. Article available at [https://www.jmir.org/2004/3/e34](https://www.jmir.org/2004/3/e34/)/; erratum available <https://www.jmir.org/2012/1/e8/>. Copyright ©Gunther Eysenbach. Originally published in the [Journal of Medical Internet](http://www.jmir.org) Research, 29.9.2004 and 04.01.2012.

This is an open-access article distributed under the terms of the Creative Commons Attribution License (<https://creativecommons.org/licenses/by/2.0/>), which permits unrestricted use, distribution, and reproduction in any medium, provided the original work, first published in the Journal of Medical Internet Research, is properly cited.
