# Supplementary material for: Safe but Lonely? Loneliness, Anxiety, and Depression Symptoms and COVID-19
Source: Front Psychol. 2020 Dec 4;11:579181. doi: 10.3389/fpsyg.2020.579181 (PMC7747668; doi:10.3389/fpsyg.2020.579181)
Supplement: Supplementary file 2 [file Data_Sheet_2.pdf]

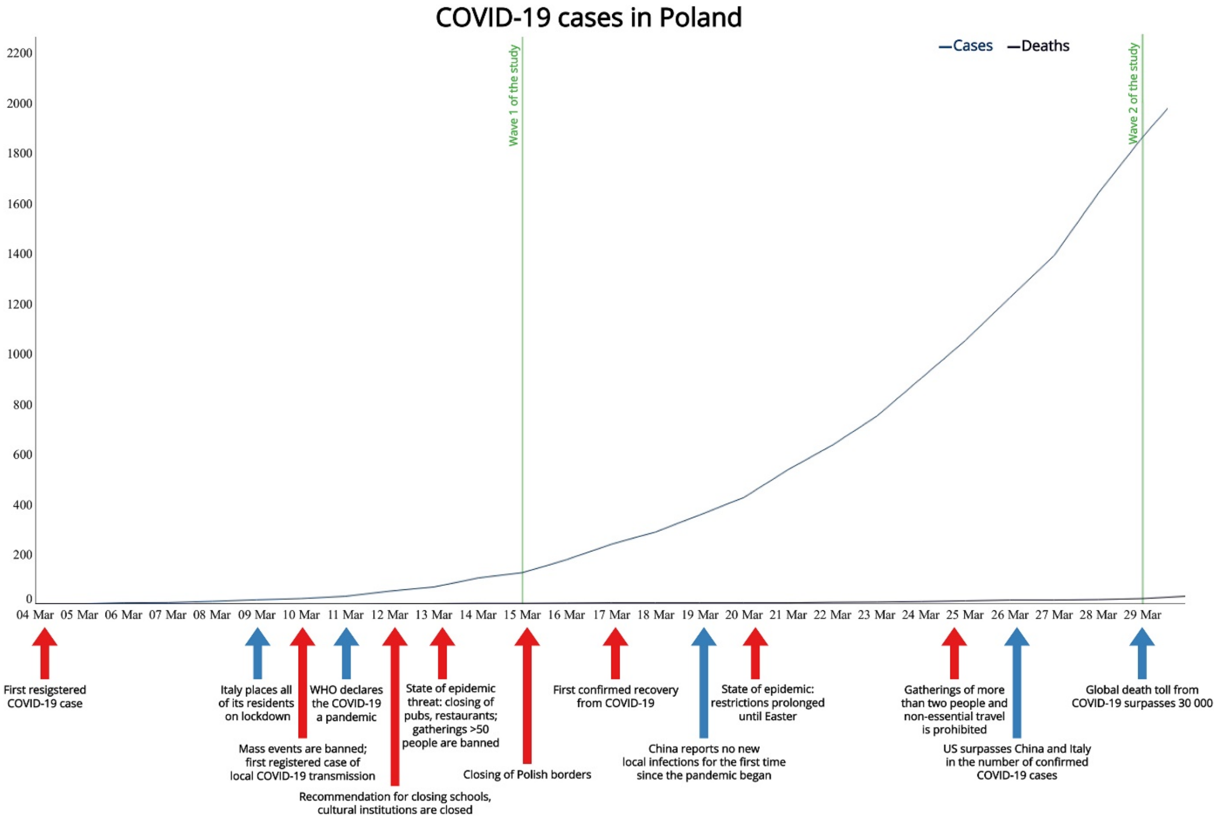

Figure S1. Timeline of COVID-19 pandemic in Poland (red arrows), world (blue arrows) overlaid on Polish COVID-19 cases and deaths curve. Green lines show the timing of each Wave of study.

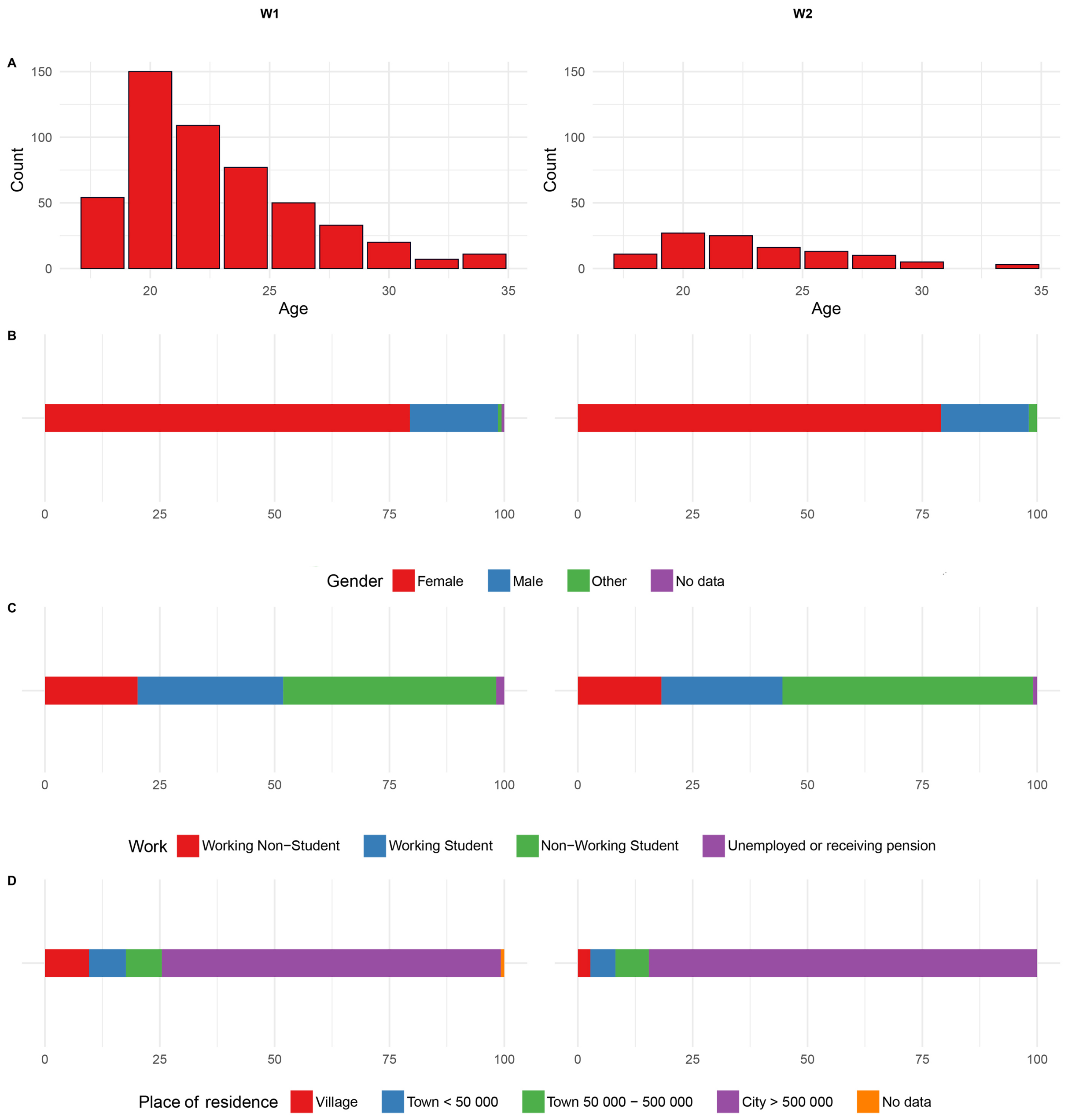

Figure S2. Basic demographic characteristics of the initial cohort of participants (W1; n = 511) and W2 (n = 110).

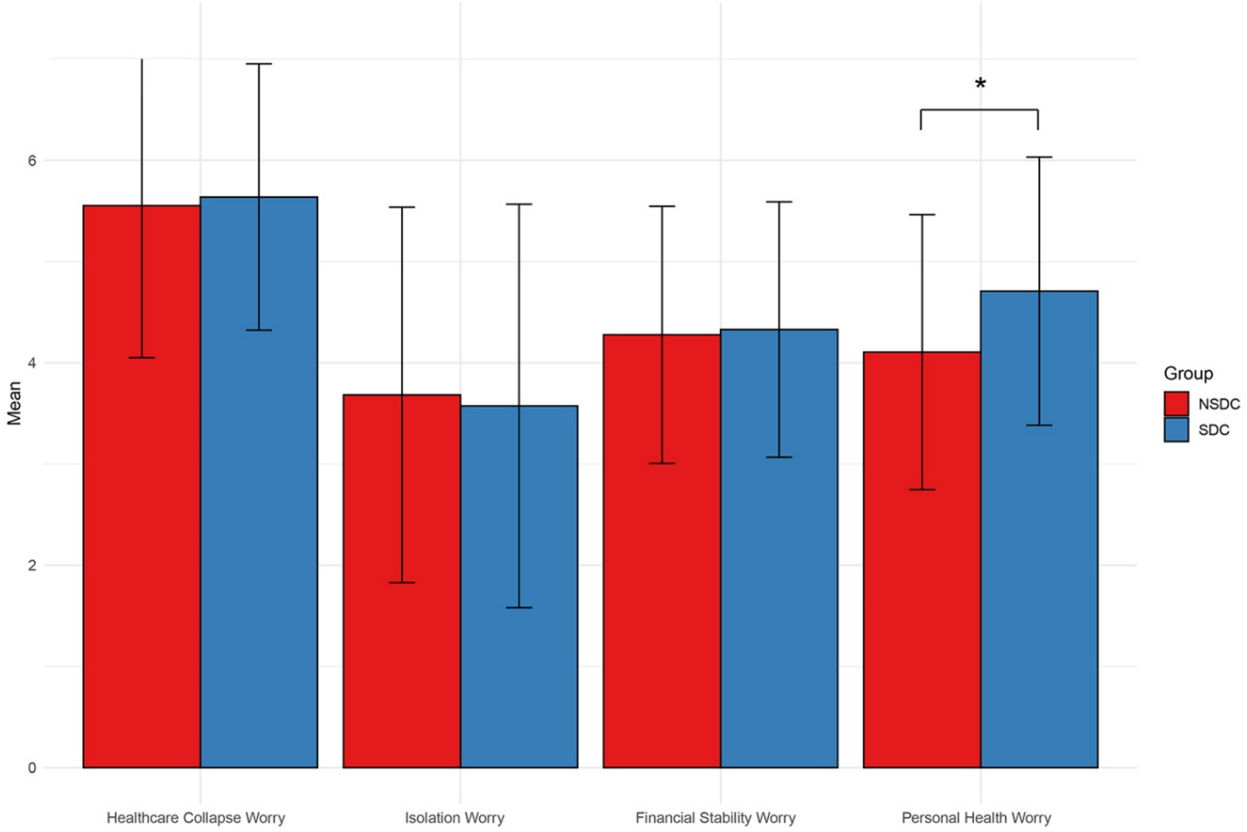

Figure S3. Affective response to specific COVID-19 issues in individuals who complied (SDC) or did not comply (NSDC) with social distancing recommendations.

COVID-19 subscales

Risk Perception: For the risk perception items, three factors with eigenvalues greater than one were obtained and explained overall 84% of response variance. Three subscales were created by choosing the items with loadings greater than .50 on each scale [Contact Risk (48%): (a) contact with somebody with COVID-19; (b) contracting the virus; (c) developing mild symptoms; Severe Symptoms Risk (21%): (a) developing severe symptoms; (b) hospitalization; Financial Problems Risk (15%): (a) losing job; (b) losing income].

Affective Response: In the same manner, affective response items were factored in four subscales explaining overall 69% of response variance [Healthcare Collapse Worry (29%), Isolation Worry (18%); Financial Stability Worry (11%); Personal Health Worry (11%)]. Healthcare Collapse Worry reflected the level to which participants were concerned about the healthcare system's ability to provide care to them and the population in general. Isolation Worry measured the level to which participants worried about the long-term impact of self-isolation on their social and psychological well-being. Financial Stability Worry was linked to the long-term COVID-19 impact on one's financial stability, access to resources, and economy in general. Finally, Personal Health Worry reflected concern about COVID-19 as a threat to personal health and the health of close ones. One item examining inability to access reliable information about the COVID-19 was not included in any affective subscale; however, as the scale as a whole showed a high level of reliability, the item was not excluded from the overall score.

Subjective Complaints: Items were factored into three subscales, which explained 64% of response variance. [Social Isolation Complaints (SIC: 44%): (a) change of normal daily activity; (b) inability to meet with family; (c) inability to meet with friends; (d) restricted contact with other people; (e) loneliness; (f) boredom; Lack of Control Complaints (LCC: 11%): (a) being overwhelmed with COVID-19 news; (b) uncertainty; (c) lack of sense of control and Nonsocial Deprivation Complaints (NDC: 9%): (a) lack of reliable information about COVID-19; (b) inability to access goods and services; (c) restricted freedom of movement]. One item (restricted freedom of movement) had loadings over 0.5 for two subscales, and thus it was included in both SIC and in NDC.
